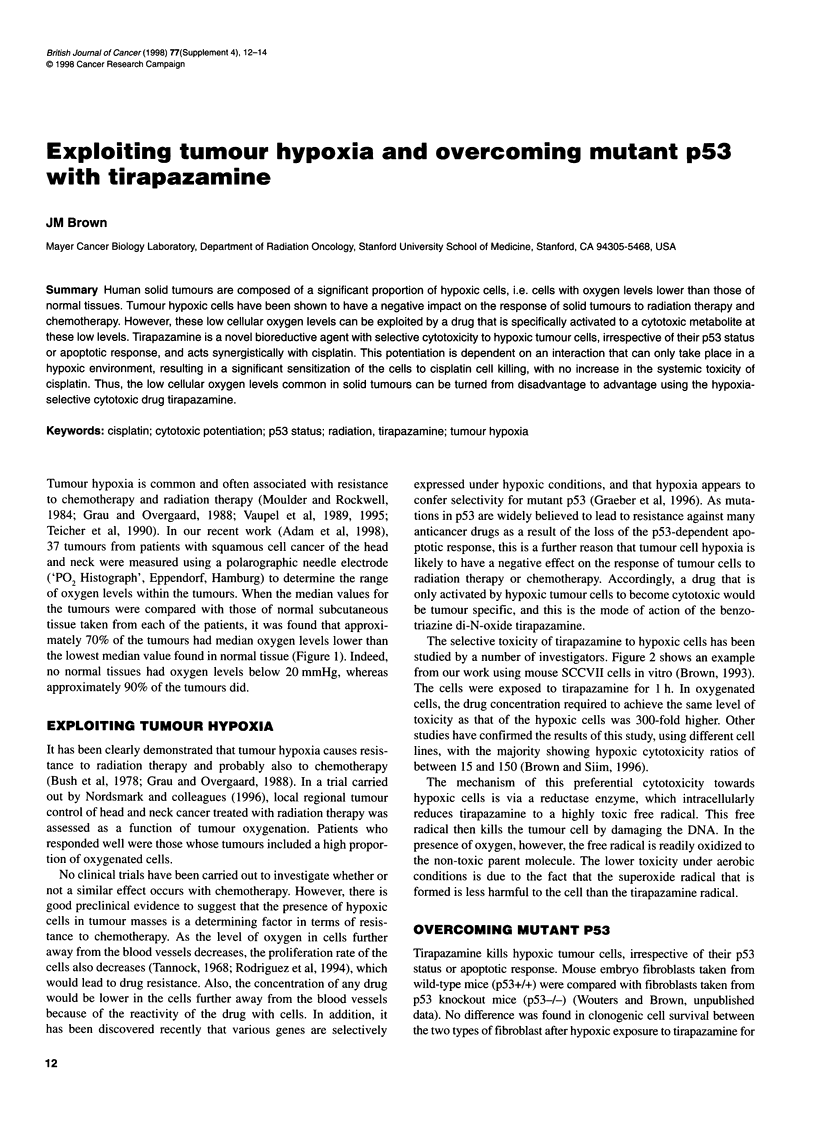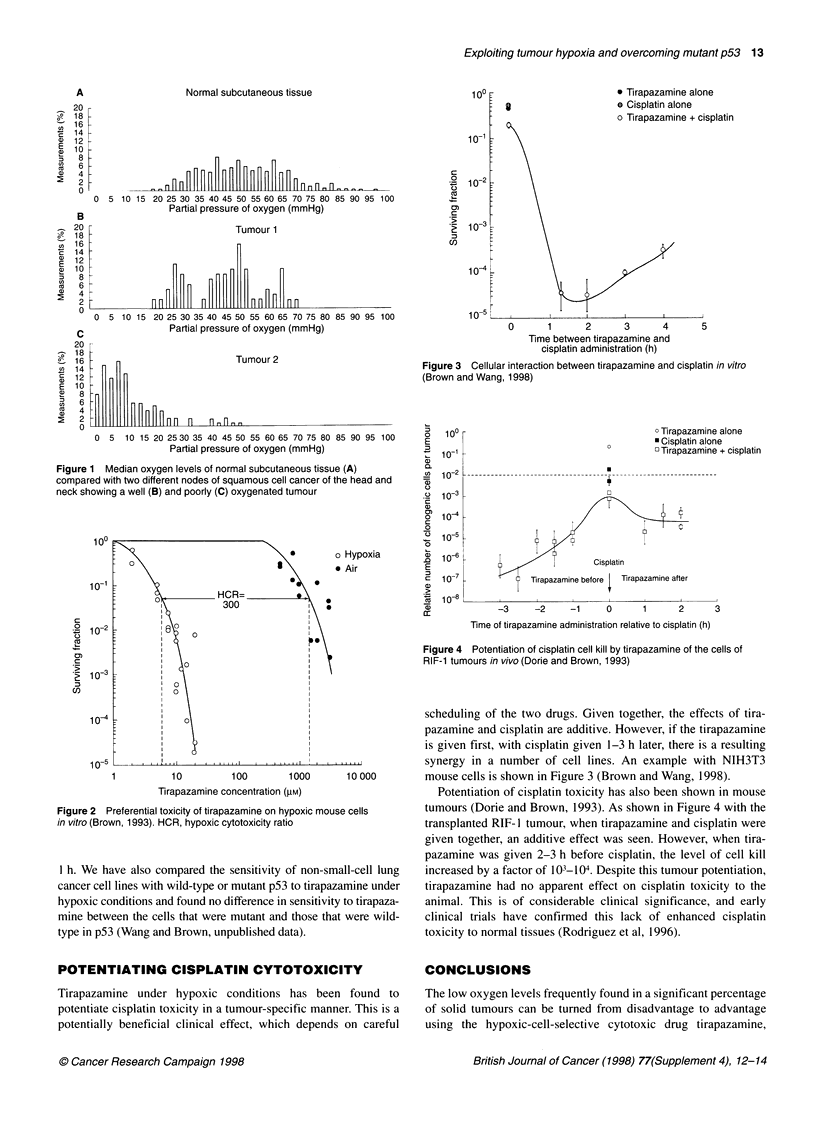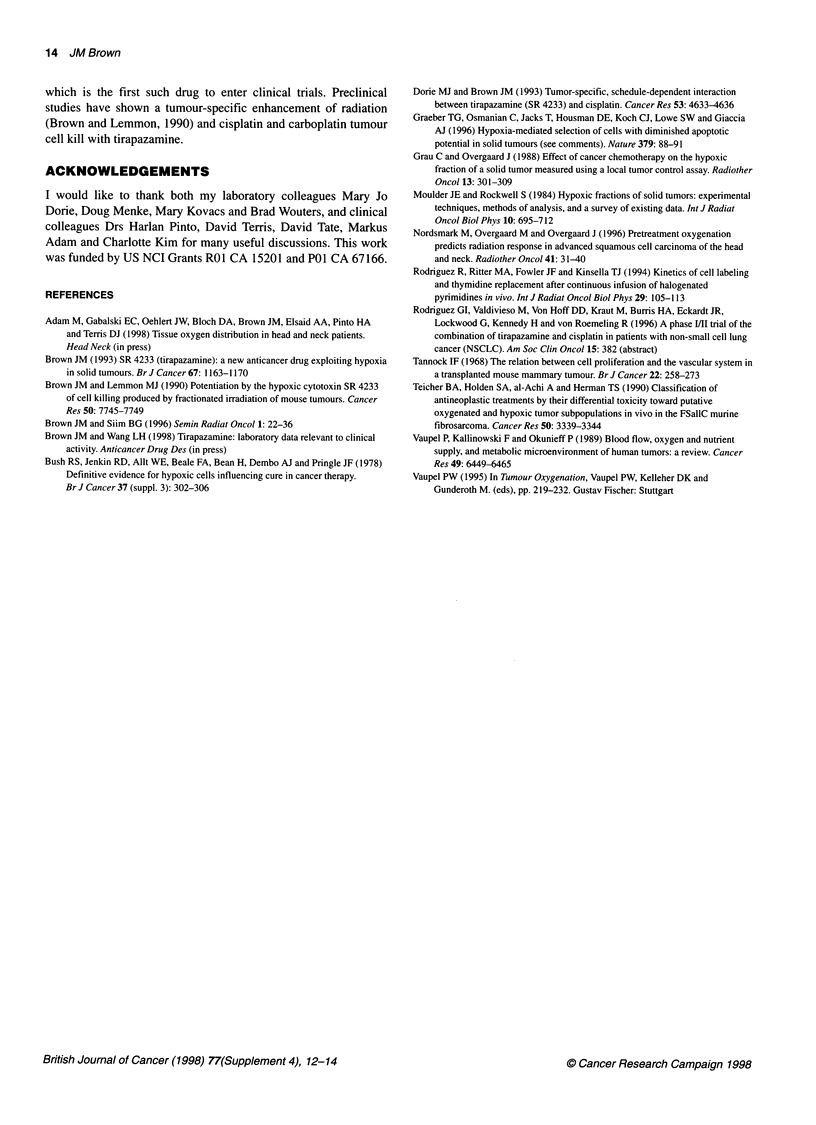# Exploiting tumour hypoxia and overcoming mutant p53 with tirapazamine.

**DOI:** 10.1038/bjc.1998.430

**Published:** 1998-06

**Authors:** J. M. Brown

**Affiliations:** Mayer Cancer Biology Laboratory, Department of Radiation Oncology, Stanford University School of Medicine, CA 94305-5468, USA.

## Abstract

Human solid tumours are composed of a significant proportion of hypoxic cells, i.e. cells with oxygen levels lower than those of normal tissues. Tumour hypoxic cells have been shown to have a negative impact on the response of solid tumours to radiation therapy and chemotherapy. However, these low cellular oxygen levels can be exploited by a drug that is specifically activated to a cytotoxic metabolite at these low levels. Tirapazamine is a novel bioreductive agent with selective cytotoxicity to hypoxic tumour cells, irrespective of their p53 status or apoptotic response, and acts synergistically with cisplatin. This potentiation is dependent on an interaction that can only take place in a hypoxic environment, resulting in a significant sensitization of the cells to cisplatin cell killing, with no increase in the systemic toxicity of cisplatin. Thus, the low cellular oxygen levels common in solid tumours can be turned from disadvantage to advantage using the hypoxia-selective cytotoxic drug tirapazamine.


British Journal of Cancer (1998) 77(Supplement 4), 12–14
© 1998 Cancer Research Campaign

### Exploiting tumour hypoxia and overcoming mutant p53 with tirapazamine

**JM Brown**

Mayer Cancer Biology Laboratory, Department of Radiation Oncology, Stanford University School of Medicine, Stanford, CA 94305-5468, USA

**Summary** Human solid tumours are composed of a significant proportion of hypoxic cells, i.e. cells with oxygen levels lower than those of normal tissues. Tumour hypoxic cells have been shown to have a negative impact on the response of solid tumours to radiation therapy and chemotherapy. However, these low cellular oxygen levels can be exploited by a drug that is specifically activated to a cytotoxic metabolite at these low levels. Tirapazamine is a novel bioreductive agent with selective cytotoxicity to hypoxic tumour cells, irrespective of their p53 status or apoptotic response, and acts synergistically with cisplatin. This potentiation is dependent on an interaction that can only take place in a hypoxic environment, resulting in a significant sensitization of the cells to cisplatin cell killing, with no increase in the systemic toxicity of cisplatin. Thus, the low cellular oxygen levels common in solid tumours can be turned from disadvantage to advantage using the hypoxia-selective cytotoxic drug tirapazamine.

**Keywords:** cisplatin; cytotoxic potentiation; p53 status; radiation, tirapazamine; tumour hypoxia

Tumour hypoxia is common and often associated with resistance to chemotherapy and radiation therapy (Moulder and Rockwell, 1984; Grau and Overgaard, 1988; Vaupel et al, 1989, 1995; Teicher et al, 1990). In our recent work (Adam et al, 1998), 37 tumours from patients with squamous cell cancer of the head and neck were measured using a polarographic needle electrode ('$PO_2$ Histograph', Eppendorf, Hamburg) to determine the range of oxygen levels within the tumours. When the median values for the tumours were compared with those of normal subcutaneous tissue taken from each of the patients, it was found that approximately 70% of the tumours had median oxygen levels lower than the lowest median value found in normal tissue (Figure 1). Indeed, no normal tissues had oxygen levels below 20 mmHg, whereas approximately 90% of the tumours did.

### EXPLOITING TUMOUR HYPOXIA

It has been clearly demonstrated that tumour hypoxia causes resistance to radiation therapy and probably also to chemotherapy (Bush et al, 1978; Grau and Overgaard, 1988). In a trial carried out by Nordsmark and colleagues (1996), local regional tumour control of head and neck cancer treated with radiation therapy was assessed as a function of tumour oxygenation. Patients who responded well were those whose tumours included a high proportion of oxygenated cells.

No clinical trials have been carried out to investigate whether or not a similar effect occurs with chemotherapy. However, there is good preclinical evidence to suggest that the presence of hypoxic cells in tumour masses is a determining factor in terms of resistance to chemotherapy. As the level of oxygen in cells further away from the blood vessels decreases, the proliferation rate of the cells also decreases (Tannock, 1968; Rodriguez et al, 1994), which would lead to drug resistance. Also, the concentration of any drug would be lower in the cells further away from the blood vessels because of the reactivity of the drug with cells. In addition, it has been discovered recently that various genes are selectively expressed under hypoxic conditions, and that hypoxia appears to confer selectivity for mutant p53 (Graeber et al, 1996). As mutations in p53 are widely believed to lead to resistance against many anticancer drugs as a result of the loss of the p53-dependent apoptotic response, this is a further reason that tumour cell hypoxia is likely to have a negative effect on the response of tumour cells to radiation therapy or chemotherapy. Accordingly, a drug that is only activated by hypoxic tumour cells to become cytotoxic would be tumour specific, and this is the mode of action of the benzotriazine di-N-oxide tirapazamine.

The selective toxicity of tirapazamine to hypoxic cells has been studied by a number of investigators. Figure 2 shows an example from our work using mouse SCCVII cells in vitro (Brown, 1993). The cells were exposed to tirapazamine for 1 h. In oxygenated cells, the drug concentration required to achieve the same level of toxicity as that of the hypoxic cells was 300-fold higher. Other studies have confirmed the results of this study, using different cell lines, with the majority showing hypoxic cytotoxicity ratios of between 15 and 150 (Brown and Siim, 1996).

The mechanism of this preferential cytotoxicity towards hypoxic cells is via a reductase enzyme, which intracellularly reduces tirapazamine to a highly toxic free radical. This free radical then kills the tumour cell by damaging the DNA. In the presence of oxygen, however, the free radical is readily oxidized to the non-toxic parent molecule. The lower toxicity under aerobic conditions is due to the fact that the superoxide radical that is formed is less harmful to the cell than the tirapazamine radical.

### OVERCOMING MUTANT P53

Tirapazamine kills hypoxic tumour cells, irrespective of their p53 status or apoptotic response. Mouse embryo fibroblasts taken from wild-type mice (p53+/+) were compared with fibroblasts taken from p53 knockout mice (p53–/–) (Wouters and Brown, unpublished data). No difference was found in clonogenic cell survival between the two types of fibroblast after hypoxic exposure to tirapazamine for

**Figure 1** Median oxygen levels of normal subcutaneous tissue (**A**) compared with two different nodes of squamous cell cancer of the head and neck showing a well (**B**) and poorly (**C**) oxygenated tumour

**Figure 2** Preferential toxicity of tirapazamine on hypoxic mouse cells *in vitro* (Brown, 1993). HCR, hypoxic cytotoxicity ratio

1 h. We have also compared the sensitivity of non-small-cell lung cancer cell lines with wild-type or mutant p53 to tirapazamine under hypoxic conditions and found no difference in sensitivity to tirapazamine between the cells that were mutant and those that were wild-type in p53 (Wang and Brown, unpublished data).

### POTENTIATING CISPLATIN CYTOTOXICITY

Tirapazamine under hypoxic conditions has been found to potentiate cisplatin toxicity in a tumour-specific manner. This is a potentially beneficial clinical effect, which depends on careful

**Figure 3** Cellular interaction between tirapazamine and cisplatin *in vitro* (Brown and Wang, 1998)

**Figure 4** Potentiation of cisplatin cell kill by tirapazamine of the cells of RIF-1 tumours *in vivo* (Dorie and Brown, 1993)

scheduling of the two drugs. Given together, the effects of tirapazamine and cisplatin are additive. However, if the tirapazamine is given first, with cisplatin given 1–3 h later, there is a resulting synergy in a number of cell lines. An example with NIH3T3 mouse cells is shown in Figure 3 (Brown and Wang, 1998).

Potentiation of cisplatin toxicity has also been shown in mouse tumours (Dorie and Brown, 1993). As shown in Figure 4 with the transplanted RIF-1 tumour, when tirapazamine and cisplatin were given together, an additive effect was seen. However, when tirapazamine was given 2–3 h before cisplatin, the level of cell kill increased by a factor of $10^3$–$10^4$. Despite this tumour potentiation, tirapazamine had no apparent effect on cisplatin toxicity to the animal. This is of considerable clinical significance, and early clinical trials have confirmed this lack of enhanced cisplatin toxicity to normal tissues (Rodriguez et al, 1996).

### CONCLUSIONS

The low oxygen levels frequently found in a significant percentage of solid tumours can be turned from disadvantage to advantage using the hypoxic-cell-selective cytotoxic drug tirapazamine,

*© Cancer Research Campaign 1998* 

which is the first such drug to enter clinical trials. Preclinical studies have shown a tumour-specific enhancement of radiation (Brown and Lemmon, 1990) and cisplatin and carboplatin tumour cell kill with tirapazamine.

### ACKNOWLEDGEMENTS

I would like to thank both my laboratory colleagues Mary Jo Dorie, Doug Menke, Mary Kovacs and Brad Wouters, and clinical colleagues Drs Harlan Pinto, David Terris, David Tate, Markus Adam and Charlotte Kim for many useful discussions. This work was funded by US NCI Grants R01 CA 15201 and P01 CA 67166.

### REFERENCES

Adam M, Gabalski EC, Oehlert JW, Bloch DA, Brown JM, Elsaid AA, Pinto HA and Terris DJ (1998) Tissue oxygen distribution in head and neck patients. *Head Neck* (in press)

Brown JM (1993) SR 4233 (tirapazamine): a new anticancer drug exploiting hypoxia in solid tumours. *Br J Cancer* **67**: 1163–1170

Brown JM and Lemmon MJ (1990) Potentiation by the hypoxic cytotoxin SR 4233 of cell killing produced by fractionated irradiation of mouse tumours. *Cancer Res* **50**: 7745–7749

Brown JM and Siim BG (1996) *Semin Radiat Oncol* **1**: 22–36

Brown JM and Wang LH (1998) Tirapazamine: laboratory data relevant to clinical activity. *Anticancer Drug Des* (in press)

Bush RS, Jenkin RD, Allt WE, Beale FA, Bean H, Dembo AJ and Pringle JF (1978) Definitive evidence for hypoxic cells influencing cure in cancer therapy. *Br J Cancer* **37** (suppl. 3): 302–306

Dorie MJ and Brown JM (1993) Tumor-specific, schedule-dependent interaction between tirapazamine (SR 4233) and cisplatin. *Cancer Res* **53**: 4633–4636

Graeber TG, Osmanian C, Jacks T, Housman DE, Koch CJ, Lowe SW and Giaccia AJ (1996) Hypoxia-mediated selection of cells with diminished apoptotic potential in solid tumours (see comments). *Nature* **379**: 88–91

Grau C and Overgaard J (1988) Effect of cancer chemotherapy on the hypoxic fraction of a solid tumor measured using a local tumor control assay. *Radiother Oncol* **13**: 301–309

Moulder JE and Rockwell S (1984) Hypoxic fractions of solid tumors: experimental techniques, methods of analysis, and a survey of existing data. *Int J Radiat Oncol Biol Phys* **10**: 695–712

Nordsmark M, Overgaard M and Overgaard J (1996) Pretreatment oxygenation predicts radiation response in advanced squamous cell carcinoma of the head and neck. *Radiother Oncol* **41**: 31–40

Rodriguez R, Ritter MA, Fowler JF and Kinsella TJ (1994) Kinetics of cell labeling and thymidine replacement after continuous infusion of halogenated pyrimidines *in vivo*. *Int J Radiat Oncol Biol Phys* **29**: 105–113

Rodriguez GI, Valdivieso M, Von Hoff DD, Kraut M, Burris HA, Eckardt JR, Lockwood G, Kennedy H and von Roemeling R (1996) A phase I/II trial of the combination of tirapazamine and cisplatin in patients with non-small cell lung cancer (NSCLC). *Am Soc Clin Oncol* **15**: 382 (abstract)

Tannock IF (1968) The relation between cell proliferation and the vascular system in a transplanted mouse mammary tumour. *Br J Cancer* **22**: 258–273

Teicher BA, Holden SA, al-Achi A and Herman TS (1990) Classification of antineoplastic treatments by their differential toxicity toward putative oxygenated and hypoxic tumor subpopulations in vivo in the FSaIIC murine fibrosarcoma. *Cancer Res* **50**: 3339–3344

Vaupel P, Kallinowski F and Okunieff P (1989) Blood flow, oxygen and nutrient supply, and metabolic microenvironment of human tumors: a review. *Cancer Res* **49**: 6449–6465

Vaupel PW (1995) In *Tumour Oxygenation*, Vaupel PW, Kelleher DK and Gunderoth M. (eds), pp. 219–232. Gustav Fischer: Stuttgart

© Cancer Research Campaign 1998